# Occurrence of *Albifimbria verrucaria* in the Blood of a Female Child With Neuroblastoma

**DOI:** 10.3389/fmed.2020.00013

**Published:** 2020-02-14

**Authors:** Riccardo Masetti, Antonio Prodi, Andrea Liberatore, Filomena Carfagnini, Eleonora Cappelletti, Davide Leardini, Andrea Pession, Elena De Carolis, Monica Cricca

**Affiliations:** ^1^“Lalla Seràgnoli”, Hematology-Oncology Unit, Department of Pediatrics, University of Bologna, Bologna, Italy; ^2^Department of Agricultural and Food Sciences, University of Bologna, Bologna, Italy; ^3^Department of Experimental, Diagnostic and Specialty Medicine, University of Bologna, Bologna, Italy; ^4^Pediatric Radiology, Sant'Orsola Malpighi Hospital, Bologna, Italy; ^5^Institute of Microbiology, IRCCS, Catholic University of the Sacred Heart, Polyclinic University Foundation Agostino Gemelli, Rome, Italy

**Keywords:** blood, neuroblastoma, immunodeficiency, invasive infection, filamentous fungus, *Albifimbria verrucaria*, climatic change adaption

## Abstract

We report for the first time the occurrence of a filamentous fungus, *Albifimbria verrucaria*, in the blood of a pediatric neuroblastoma patient. The *Albifimbria* genus comprises common soil-inhabiting and saprophytic fungi and has been isolated as a plant pathogen in Northern and Southern Italy. As a human pathogen, *A. verrucaria* has been implicated in keratitis and can produce trichothecene toxins, which are weakly cytotoxic for mammalian cell lines. *A. verrucaria* was isolated from blood during the follow-up of a previous coagulase-negative *Staphylococcus* catheter-related infection. Lung nodules, compatible with fungal infection, had been observed on a CT scan 6 months earlier; they still persist. Possible routes of transmission were considered to be airborne, catheter related, or transfusion dependent, as the patient had undergone platelet and red blood cell transfusions during rescue chemotherapy. No filamentous fungi were isolated from sputum or CVCs. In conclusion, we describe an unprecedented fungemia caused by *A. verrucaria* and show how an unexpected pathogen may be acquired from the environment by patients at high risk due to immunosuppression. The route of transmission remains unknown.

## Introduction

Invasive fungal infections are an emerging problem worldwide. Hyaline molds, such as *Fusarium* and *Scedosporium* spp., are the filamentous fungi which can most frequently cause fungemia in humans (1). The occurrence of invasive infections is predominantly driven by the increasing use of invasive devices such as central venous catheters (CVC), especially in immunocompromised patients. Another critical factor is the emergence of saprophytic environmental fungi and their acquisition of thermotolerance, due to global warming, which is a key step toward opportunistic human infections (2, 3). Emerging pathogens are sometimes resistant to the antifungals available, increasing the threat of new fungal diseases (4). *Albifimbria verrucaria* is a saprophytic fungus that belongs to the *Stachybotryaceae* family, found mainly in soil and as a plant pathogen. More than 30 species of *Albifimbria* have been described worldwide (5), and occurrence of itraconazole-tolerant species in soil and food products has been reported. The importance of *A. verrucaria* in humans arose from the observation that it produces a potent mycotoxin, which may produce numerous adverse effects, such as the inhibition of protein synthesis, immune suppression, and impairment of alveolar macrophage function (6). Some authors have recently reported *Myrotecium* spp., also called *Albifimbria* spp., as the causative agents of keratitis in immunocompetent patients (7). To our knowledge, ours is the only report of *A. verrucaria* as a causative agent of infection in humans. Here, we report, for the first time, the occurrence of *A. verrucaria* in the blood of a child with neuroblastoma (NBL).

## Case Presentation

**Figure 1:**
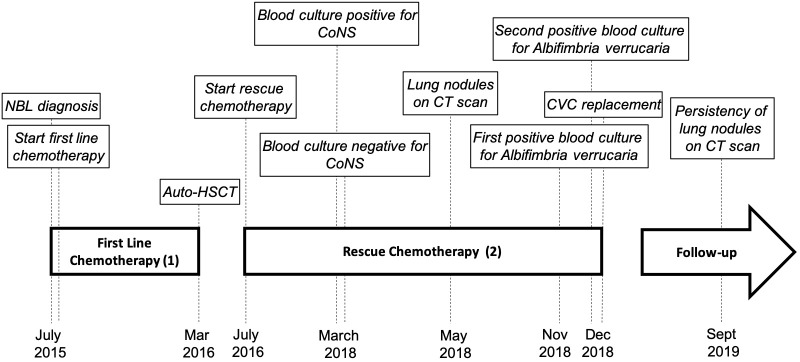
Patient medical history timeline. **(1)** First-line chemotherapy: SIOPEN NB-HR-01 protocol consisting of 10 weeks of induction chemotherapy (COJEC scheme), surgery on the primary tumor, auto HSCT with Busulfan and Melphalan, 21 Gys of radiotherapy, and six courses of anti-GD2 antibody. **(2)** Rescue therapy: temozolamide 100 mg/m^2^ and Irinotecan 50 mg/m^2^ for five consecutive days every 3 weeks. The patient received 36 courses of chemotherapy and several blood and platelet transfusions due to the myelotoxicity of the chemotherapy. CoNS, coagulase-negative *Staphylococcus* (*Staphylococcus haemolyticus*).

**Figure 2:**
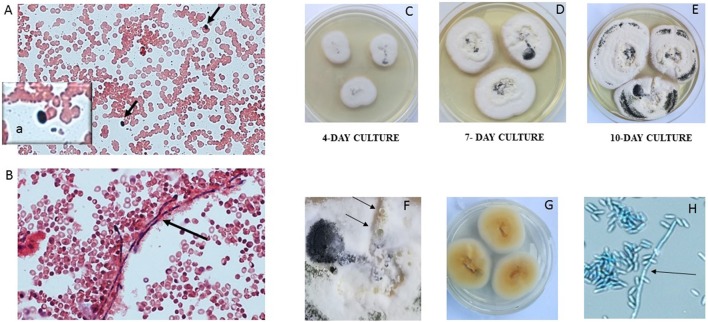
Presence of conidia (**A**, black arrows and inset a) and hyphae (**B**, black arrow) in the blood culture (20× magnification, Gram stain). The mold isolated from blood culture was subcultured on Sabouraud dextrose at 30°C. *A. verrucaria* colonies were flat, smooth, moist, white to cream, with regular and sharp margins (**C–E** growth over 10 days). The colonies developed a black pigment represented more and more along the days of culture and produced serous vesicles (**F**, black arrows). The reverse was yellow to orange **(G)**. Septate hyphae (**H**, black arrow) and grapes of conidia **(H)** were present on microscopic examination (lactophenol cotton blue, 40× magnification).

**Figure 3:**
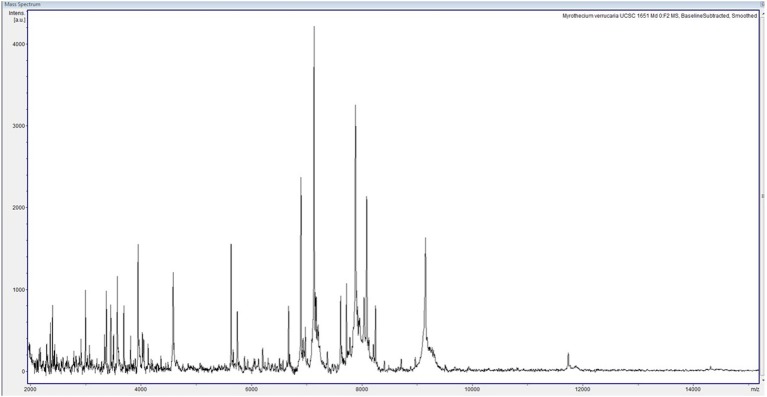
Spectrum of *Albifimbria verrucaria* collected within a mass range of 2,000–20,000 Da. The spectrum was analyzed with Bruker Biotyper 3.1 software and compared with those of the reference database.

We report the case of a female child diagnosed with high-risk NBL at the age of 2 years and 5 months (Figure 1). Written informed consent was obtained from the legal representatives of the patient for the publication of this case report. At disease onset, the primary lesion was a left adrenal mass associated with multiple bone secondary localizations and bone marrow infiltration. She was treated according to the SIOPEN NB-HR-01 protocol (8), which consists of induction chemotherapy, peripheral blood stem cell harvest, attempted complete excision of the primary tumor, myeloablative therapy followed by peripheral blood stem cell rescue, radiotherapy, and immunotherapy. The patient was resistant to this first-line chemotherapy protocol, so she was given rescue treatment of Temozolamide and Irinotecan (9). She received a 5-day course of chemotherapy every 3 weeks for 18 months. Owing to the side effects of the chemotherapy, she became severely immunocompromised and transfusion dependent, receiving at least two to three filtered and irradiated erythrocyte and platelet transfusions per month. During this 18-month period, at the age of 5, she experienced an episode of fever and chills at the end of a chemotherapy infusion. Blood samples were collected and sent to the Microbiology Lab, Sant'Orsola Malpighi Hospital, Bologna, Italy, for routine diagnostic procedures. Coagulase-negative *Staphylococcus* was isolated from one of the lines of the CVC; the other was negative. Concomitant blood analysis showed a slight increase in C-reactive protein (CRP) (6.62 mg/dl). Suspecting a CVC-related infection, both lines were closed, and lock therapy with 0.8 ml of a solution of 16 mg of Vancomycine and 1,613 UI of Urokinase was administered. After treatment, the cultures of blood drawn from the CVC and peripheral blood became negative. Over the following 6 months, she had sporadic episodes of fever without any other clinical signs with a variable increase in C-reactive protein (maximum value, 11.32 mg/dl). In consideration of these clinical findings, the blood cultures from the CVC lines were serially repeated every 3 weeks before the beginning of chemotherapy. At the age of 5 years and 9 months, a filamentous fungus was isolated from two serial CVC blood cultures, one collected from each of the lines over a 2-week interval. The blood was inoculated into BD BACTEC™ Peds Plus™ media and incubated in the Bactec FX Instrument. On direct microscopic examination of blood cultures, we observed the presence of conidia and septate hyphae (time to positivity was ~52 h for the first blood culture and 26 h for the second). After 2 days of incubation on horse blood agar and Sabouraud Chloramphenicol agar tubes (Vakutainer Kima, Padova, Italy) at 30°C, a slow-growing mold appeared on both media (Figure 2). Identification was not possible with matrix-assisted laser desorption/ionization time of flight analysis (MALDI Biotyper Instrument, Bruker Daltonics, Bremen, Germany) because the spectrum of *A. verrucaria* (Figure 3) was not present in the filamentous fungi reference database, which includes 430 mass spectrum profiles developed at the Polyclinic Gemelli Hospital (10). Thus, sequencing was used to identify the mold. Total genomic DNA was extracted with Dneasy Plant Mini Kit (Qiagen, Milan, Italy). First, the isolate was mechanically broken down with glass beads (G8772-100G, Sigma-Aldrich, Milan, Italy) and bead beater; then, it was treated according to the manufacturer's instructions. Part of the β-tubulin (Tub) gene was amplified using primers BT-2a/BT-2b (11); the sequences obtained showed 99.06% agreement with the extype of *A. verrucaria* CBS 328.52 (GeneBank KU845969.1) and 100% with *A. verrucaria* strain CBS 189.46 (GeneBank KU845965.1). Nucleotide sequences of the isolate were deposited in the GeneBank database under the accession number (GeneBank MN428067). Sequencing with ITS1-4 primers was not conclusive for the identification at species level.

Finally, we performed antifungal susceptibility testing using the commercial microdilution method Sensititre YeastOne (SYO ITAMYUCC, Thermo Scientific, Italy), following the manufacturer-recommended protocol (12). After 2 days of incubation at 30°C, we found the following minimum inhibitory concentrations (MIC): amphotericin B, 0.25 μg/ml; posaconazole, 0.5 μg/ml; isavuconazole, 0.5 μg/ml; voriconazole, 0.5 μg/ml; itraconazole, 2 μg/ml; fluconazole, 256 μg/ml; and echinocandins (Caspofungin, Anidulafungin and Micafungin), >8 μg/ml. We also tested some drugs (Voriconazole, Posaconazole, Itraconazole) with the reference method for conidia-forming molds EUCAST (definitive document E.DEF 9.3.), and at 30°C, the same MIC values were obtained.

Shortly after finding the second positive blood culture, the CVC was replaced, as it was the suspected source of fungemia. The catheter was treated with dithiothreitol (Thermo Fisher, Italy), the solution seeded into Sabouraud-CAF agar tubes as well as brain heart infusion broth (Biolife, Italy), and incubated for 3 and 2 weeks, respectively. No fungal or bacterial growth was observed. Thus, a catheter-related infection has been excluded.

**Figure 4:**
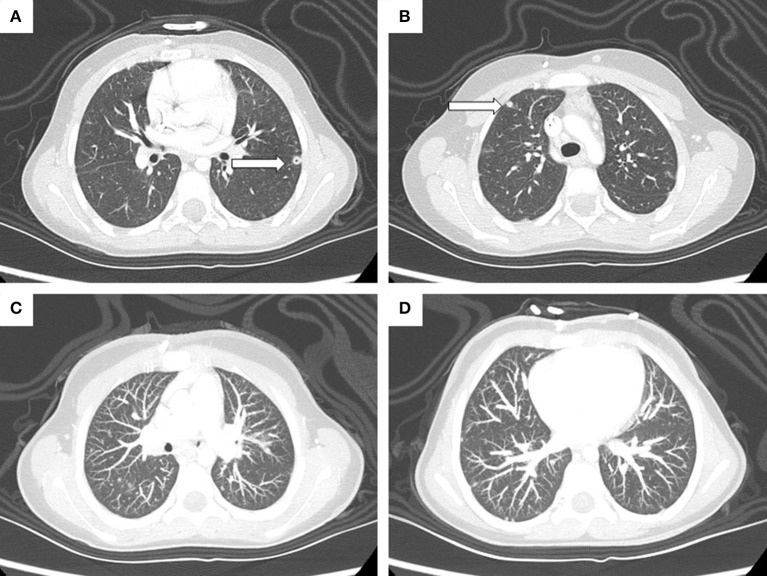
High-resolution CT scan highlights a 6-mm cavitated nodule (arrow) in the anteromedial segment of the left inferior lobe **(A)** and a 5-mm subpleural nodule (arrow) in the anterior segment of the right superior lobe **(B)**. Maximum intensity projection (MIP) reconstruction image **(C,D)** shows the presence of multiple disseminated parenchymal and subpleural nodules (max. 5 mm).

Routine abdomen and chest CT scans were performed during rescue therapy to follow-up treatment of the NBL masses. These revealed multiple small bilateral lesions, mainly at the bases, with a nodular shape and ground glass features (Figure 4). However, the patient did not present any concomitant respiratory symptoms. The lung is not a preferential site for NBL metastases (3.3%) (13), and the diameter of the lesions was not influenced by chemotherapy. Hence, after two positive blood cultures, we hypothesized the involvement of *A. verrucaria* in the pathogenesis of the lung lesions. To test this hypothesis, we cultured a sputum specimen, but no fungus was isolated. No invasive procedures have been performed, and no antifungal therapy has been administered, as the patient has not presented any respiratory or systemic clinical signs or symptoms. Furthermore, the patient has received very high doses of chemotherapy, and both the antifungal therapy and invasive procedure to obtain a byoptic sample could produce more side effects than in a healthy subject. The patient is now being followed up at our clinic for treatment of NBL, as the disease is still in progress and she is waiting to receive more radiotherapy.

## Discussion

To our knowledge, this is the first case of *A. verrucaria* in an immunocompromised child with NBL. *A. verrucaria* belongs to the *Stachybotryaceae* family. Members of this family include important plant and human pathogens, as well as several species used industrially and commercially as biodegraders and biocontrol agents (14). Some species of *Albifimbria* have been isolated in extreme environments such as hot desert sands from Saudi Arabia and Jordan (15). In Northern and Southern Italy, *A. verrucaria* has been described as a causative agent of severe leaf necrosis and plant decay and has recently been observed on leafy vegetable crops such as spinach, lamb's lettuce, and wild rocket (16). Its diffusion as a plant pathogen in Italy may increase the risk of opportunistic infections in immunocompromised hosts. Some authors claim that the recent emergence of environmental pathogens due to global warming and the acquisition of thermotolerance may represent a threat for human health, especially for patients with weakened immune systems (2).

*A. verrucaria* was isolated from our patient's blood while monitoring a prior coagulase-negative *Staphylococcus* catheter-related infection. Lung nodules compatible with fungal infection had been visible on CT scans 6 months earlier and still persist. We hypothesized that our patient had a primary infection in the respiratory tract and that the fungus had invaded the bloodstream. To test this hypothesis, we analyzed a sputum specimen which was negative for mold infection and all other microorganisms. We speculated that the catheter could be another possible route of transmission, but CVC culture examination was negative, so this route was excluded. The presence of *A. verrucaria* in the blood could be due to the transfusions carried out during rescue chemotherapy over an 18-month period, even though transfused blood is filtered and irradiated. Inoculation by drug infusion cannot be excluded either. No cases of *A. verrucaria* have been described in human pathology, except for two cases of keratitis in immunocompetent hosts. These patients were treated with topical natamycin and voriconazole. One patient completely recovered, but the other did not, suggesting potential resistance to azoles (7). In our case, *A. verrucaria* was resistant to fluconazole and echinocandins, and elevated MIC values for itraconazole were observed. The recent observation of azole-resistant strains of environmental *Aspergillus* spp. due to fungicide use in agricultural settings are in line with our findings (17).

So far, our patient has not developed invasive infections in deep organs, even though she has not received antifungal therapy. This may be due to the weak pathogenic potential of *A. verrucaria*, or its poor adaptation to human body temperature, in the case of direct inoculation into the blood via medical products. This report highlights not only the emergence of unusual pathogens in human immunocompromised hosts but also the need to improve diagnostic procedures and *in vitro* antifungal sensitivity testing for the prompt identification and management of fungal infections in immunocompromised hosts.

## Data Availability Statement

All datasets generated for this study are included in the article.

## Ethics Statement

The studies involving human participants were reviewed and approved by Sant'Orsola Malpighi Hospital Ethics Committee. Written informed consent to participate in this study was provided by the participants' legal guardian/next of kin. Written informed consent was obtained from the individual(s), and minor(s)' legal guardian/next of kin, for the publication of any potentially identifiable images or data included in this article.

## Author Contributions

RM and MC: conception and design of the work. RM, APr, AL, FC, EC, DL, ED, and MC: data collection. RM, APr, ED, and MC: data analysis and interpretation, manuscript writing, and critical revision of the article. RM, APe, AL, FC, EC, DL, ED, and MC: approval of the final version of the article.

### Conflict of Interest

The authors declare that the research was conducted in the absence of any commercial or financial relationships that could be construed as a potential conflict of interest.
